# Characterization of the complete chloroplast genome of *Clematis potaninii* (Ranunculaceae), a medicinal and ornamental plant

**DOI:** 10.1080/23802359.2022.2097023

**Published:** 2022-07-12

**Authors:** Ruirui Zhang, Qi Wang, Sha Yang, Zhuangzhuang Huang, Ping Wang, Yufei Liao, Xueyan Zhao

**Affiliations:** a School of Pharmacy, Shaanxi Institute of International Trade and Commerce, Xi’an, China; b Xi’an Botanical Garden of Shaanxi Province (Institute of Botany of Shaanxi Province), Xi’an, China

**Keywords:** Chloroplast genome, *Clematis potaninii*, phylogenetic tree

## Abstract

*Clematis potaninii* Maxim. is an important medicinal and ornamental plant. The length of *C. potaninii* chloroplast genome was 159,691 bp, with a large single-copy region of 79,503 bp, a small single-copy region of 18,106 bp, and two inverted repeat regions of 31,041 bp each. The chloroplast genome contains 138 genes including 94 protein-coding, eight rRNA, and 36 tRNA genes. Phylogenetic analysis showed that *C. potaninii* is closely related to *C. alternata*.

*Clematis potaninii* Maxim. (1890), which belongs to the genus *Clematis* (Ranunculaceae), is a vine plant with important medicinal and ornamental value (Flora of China Editorial Committee 1980). *Clematis* plants are widely distributed in China and have anti-inflammatory and analgesic, antioxidation, and antitumor activities (Wei et al. 2017; Li et al. 2018). *Clematis* plants are known as ‘queen of climbing plants,’ and *C. potaninii* is an excellent greening plant because of its thick foliage and large colorful flowers (Wang et al. 2020). To contribute to the bioinformatics and systematics of this taxon, high-throughput sequencing analysis was performed.

*C. potaninii* leaves were collected from Zhuqueshan National Forest Park (33°79′N, 108°58′E; Shaanxi, China), and the voucher specimen (ZR190701) was deposited in the Shaanxi Institute of International Trade and Commerce Herbarium (Zhang R, rui09117@163.com). The collection of *C. potaninii* was in accordance with the guidelines of the Shaanxi Institute of International Trade and Commerce and China. The total genomic DNA was extracted from leaves through a modified CTAB method. Subsequently, the DNA library with an insert size of 270 bp was prepared and then sequenced on an Illumina HiSeq X Ten platform.

The obtained sequencing data were assembled using published chloroplast genomes and the default settings by GetOrganelle v1.7.2 (Jin et al. 2020). The genome was annotated using CPGAVAS2 with 2544 published plastomes as references (Shi et al. 2019). The chloroplast genome sequence was deposited into the NCBI Genbank (accession number: MW542990).

The chloroplast genome was 159,691 bp in length, with a large single copy (LSC, 79,503 bp), a small single copy (SSC, 18,106 bp), and two inverted repeats (IRa and IRb; 31,041 bp each). The overall GC content was 38.0% (LSC, 36.3%; SSC, 31.4%; and IRs, 42.0%), and the chloroplast genome contained 138 genes, with 94 protein-coding, 8 rRNA, and 36 tRNA genes.

To investigate the phylogenetic relationship of *C. potaninii*, a maximum likelihood tree was constructed using IQTREE-2.1.2 with TVM + F + R6 model (Lam-Tung et al. 2015) and the bootstrap replicates were 1000 (Figure 1). The results indicated that *C. potaninii* is closely related to *C. alternata*, forming a clade.

**Figure 1.**
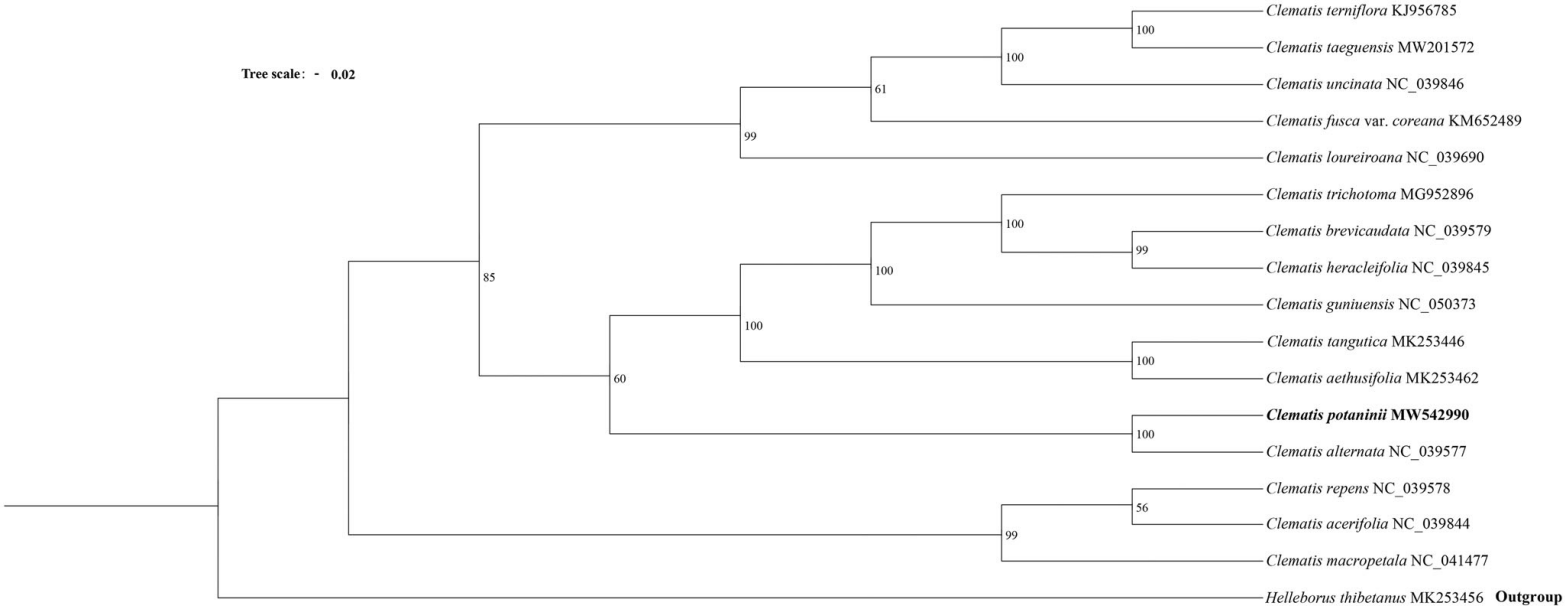
Maximum likelihood tree based on 16 complete chloroplast genome sequences of *Clematis.*

## Author contributions

Ruirui Zhang: design and drafting. Qi Wang, Sha Yang, and Zhuangzhuang Huang: collecting plant material. Ping Wang and Yufei Liao: analysis. Xueyan Zhao: analysis and drafting. All authors agree to be accountable for all aspects of the work.

## Data Availability

The genome sequence data that support the findings of this study are openly available in GenBank of NCBI (https://www.ncbi.nlm.nih.gov/) under the accession no. MW542990. The associated BioProject, SRA, and BioSample numbers are PRJNA713968, SRP310620, and SAMN18275854, respectively.
